# Anti-HMGB1 Monoclonal Antibody Ameliorates Immunosuppression after Peripheral Tissue Trauma: Attenuated T-Lymphocyte Response and Increased Splenic CD11b^**+**^Gr-1^**+**^ Myeloid-Derived Suppressor Cells Require HMGB1

**DOI:** 10.1155/2015/458626

**Published:** 2015-01-29

**Authors:** Xiangcai Ruan, Sophie S. Darwiche, Changchun Cai, Melanie J. Scott, Hans-Christoph Pape, Timothy R. Billiar

**Affiliations:** ^1^Department of Anesthesiology, First Municipal People's Hospital of Guangzhou, Affiliated Hospital of Guangzhou Medical College, Guangzhou, China; ^2^Department of Surgery, University of Pittsburgh, University of Pittsburgh Medical Center, Suite F1281, 200 Lothrop Street, Pittsburgh, PA 15213, USA; ^3^Department of Orthopaedic and Trauma Surgery, Aachen University Hospital, Pauwelsstraße 30, 52074 Aachen, Germany

## Abstract

Although tissue-derived high mobility group box 1 (HMGB1) is involved in many aspects of inflammation and tissue injury after trauma, its role in trauma-induced immune suppression remains elusive. Using an established mouse model of peripheral tissue trauma, which includes soft tissue and fracture components, we report here that treatment with anti-HMGB1 monoclonal antibody ameliorated the trauma-induced attenuated T-cell responses and accumulation of CD11b^+^Gr-1^+^ myeloid-derived suppressor cells in the spleens seen two days after injury. Our data suggest that HMGB1 released after tissue trauma contributes to signaling pathways that lead to attenuation of T-lymphocyte responses and enhancement of myeloid-derived suppressor cell expansion.

## 1. Introduction

Severe trauma can cause a profound imbalance in immune function and predispose the injured host to opportunistic infections. In this respect, nosocomial infections are a common clinical problem in trauma intensive care units in injured patients and are associated with increased morbidity, length of hospital stay, and mortality [1]. A number of studies have demonstrated that splenocyte dysfunction often observed as T-lymphocyte dysfunction and T-helper 1 (Th1) depression are associated with increased susceptibility to severe infection and/or multiple organ dysfunction (MODS) after trauma [2–4].

Myeloid-derived suppressor cells (MDSCs), a heterogeneous CD11b^+^Gr-1^+^ cell population consisting of immature myeloid cells and myeloid progenitor cells that can suppress T-cell responses by a variety of mechanisms [5, 6], have recently been associated with the profound immunosuppression seen in sepsis and trauma. Ochoa and colleagues [7, 8] found that a marked increase of CD11b^+^Gr-1^+^ MDSCs is associated with suppressed T-cell functions via the production of arginase-1 in the mouse spleen at 24 h after surgical trauma. However, under septic conditions, Moldawer and colleagues found that a different response where CD11b^+^Gr-1^+^ MDSCs expanded in the spleen, in the bone marrow, and in the peripheral lymph nodes by 3 days after sepsis and several approaches to prevent the expansion of these cells deteriorated the immunosuppression and worsened outcomes [9, 10]. The relatively different roles of MDSCs in sepsis and trauma thus warrant a characterization of CD11b^+^Gr-1^+^ MDSCs in different lymphoid compartments in traumatized mice over time.

High mobility group box nuclear protein 1 (HMGB1) is a nuclear DNA binding protein that has been shown to be a key late mediator in sepsis [11] and an early trigger of sterile inflammation in severe trauma when released from cells [12, 13]. Although HMGB1 released as the result of tissue injury or stress is involved in many aspects of inflammation, it is unknown whether HMGB1 mediates trauma-associated immune suppression.

HMGB1 has multiple functions in the regulation of immunity and inflammation, and studies have shown that HMGB1 has variable effects on T-cell responses depending on dose, redox status, and disease setting [14, 15]. Furthermore HMGB1 has been demonstrated to lead to MDSC expansion after surgical trauma in the setting of cancer [16]; however, HMGB1 has been shown to bind to and enhance the cytokine-induced effect of several inflammatory mediators, such as IL-1 [17], IL-6 [18], and TNF-*α* [19], which themselves have been shown to drive the expansion of MDSC [20, 21].

In this study, we evaluated the role of HMGB1 in trauma-associated immune suppression through examination of the contribution of HMGB1 to MDSC expansion and splenocyte responses after peripheral tissue trauma. Systemic neutralization of HMGB1 was achieved with administration of anti-HMGB1 monoclonal antibody, 2G7 after injury. Using a murine trauma model, the pseudofracture (PF) model, immune responses were assessed at specific time points out to 3 days from injury.

## 2. Materials and Methods

### 2.1. Animals

Mice used in the experimental protocols were housed in accordance with University of Pittsburgh (Pittsburgh, PA) and National Institutes of Health (NIH, Bethesda, MD) animal care guidelines in specific pathogen-free conditions. A total of 175 male C57BL/6 mice were used, aged 8–12 weeks, weighing 20–30 g, and were obtained from Charles Rivers Laboratories International (Wilmington, Mass). The animals were maintained in the University of Pittsburgh Animal Research Center with a 12 h light-dark cycle and free access to standard laboratory food and water. All animals were acclimatized for 7 days prior to being used and fasted for 12 h prior to experimental manipulation.

### 2.2. Systemic Neutralization of HMGB1

Mice were injected s.c. with a total volume of 200 *μ*L of solution containing 40 *μ*g of a mouse monoclonal anti-HMGB1 antibody 2G7 (IgG2b, noncommercial antibody, gift from Dr. K. Tracey) right after and at 24 h after the pseudofracture (PF) trauma model. Control mice received equivalent amounts of inactive control antibodies (IgG2b mAb).

### 2.3. Pseudofracture (PF) Trauma Model

PF is a model of peripheral tissue trauma, which incorporates all the elements of this injury type including the soft tissue and fracture components [22, 23]. As described previously [22, 23], donor mice were euthanized with overdoses of isoflurane (Abbott Laboratories, Chicago, Ill), and the long bones of the lower extremities were harvested sterilely. To obtain standardized concentrations, two femurs and two tibias were removed from collected tissues and crushed using a sterile mortar and pestle in the biological safety hood. The crushed bone fragments were resuspended with 2 mL phosphate-buffer solution (PBS), crushed again, and kept in a sterile tube on ice. Recipient animals were anesthetized with i.p. 70 mg/kg sodium pentobarbital (Hospira, Inc., Lake Forest, IL, for OVATION Pharmaceuticals, Deerfield, IL) and inhaled isoflurane, and both thighs were squeezed with a hemostat for 30 seconds to induce a soft tissue injury. Then, 0.15 mL of bone components suspension prepared above was injected using an 18 G needle in the area of the soft tissue injury of each thigh. After surgery and recovery from anesthesia, animals were given s.c. buprenorphine (0.10 mg/kg, Bedford Laboratories, Bedford, OH) to minimize discomfort. Anaesthetized mice that received no experimental manipulation were used as uninjured controls.

At different time points (1, 6, 24, 48, and 72 h) after PF, mice were sacrificed with overdoses of isoflurane (Hospira, Lake Forest, IL, USA), cardiac puncture was directed and performed to collect whole blood, and spleens and bone marrow were collected aseptically for further analysis.

### 2.4. Splenocyte Preparation

Splenocytes were isolated and the red blood cells were lysed by exposure for 5 min to 2 mL of red blood cell lysis buffer (Sigma, St. Louis, MO). After washing the cells twice with complete RPMI 1640, isolated splenocytes were diluted to appropriate concentrations, detailed as follows, in complete RPMI 1640 medium (Lonza, Walkersville, MD, USA), which contained 10% fetal bovine serum (FBS, Sigma), 2 mM glutamine (GIBCO Laboratories, Grand Island, NY), 1% penicillin-streptomycin (GIBCO Laboratories), and 0.05 mM 2-mercaptoethanol (2-ME, Sigma). Cell concentrations and total cell yields were assessed using Trypan blue and a hemocytometer.

### 2.5. Flow Cytometric Analysis

A 100 *μ*L aliquot of whole blood and bone marrow was lysed with 2 mL of lysing buffer. Whole blood, splenocyte, and bone marrow suspension were then blocked with 1 *μ*g/mL Fc blocker (BD Pharmingen, Mississauga, ON, Canada) and stained with appropriately diluted antibodies directly conjugated with FITC or PE according to the standard procedure and followed by fixation in 1% paraformaldehyde. Antibodies used for staining were the following: FITC-labeled anti-mouse CD11b and PE-labeled Gr1 (BD Pharmingen). All staining procedures were conducted on ice. Fluorescence was measured using Guava EasyCyte 8 flow cytometer (Millipore, Hayward, CA), and data analysis was performed using ExpressPro 8.1 software (Millipore).

### 2.6. Cell Proliferation Assay

The ability of the splenocyte cultures to produce cytokines in response to a mitogenic challenge was assessed by incubating total splenocyte cultures (5 × 10^6^ cells/mL) for 48 h (at 37°C, 5% CO_2_, and 90% humidity) in the presence of 0.25 *μ*g/mL concanavalin A (con A, Perkin Elmer Life Science, Boston, MA, USA) or 0.1 *μ*g/mL anti-CD3 mAb (clone 145-2C11, BD Pharmingen, San Diego). After that period, the supernatants were harvested, aliquoted, and stored at −20°C until being assayed for cytokines. A second portion of the splenocyte suspension was placed in a 96-well U-bottomed plate (Becton Dickinson, Franklin Lakers, NJ, USA) in aliquots of 100 *μ*L. The cells' ability to proliferate in response to mitogenic stimulation with 0 (negative control), 0.25 *μ*g/mL con A, or 0.1 *μ*g/mL anti-CD3 mAb was determined by incubation for 48 h at 37°C in a 5% CO_2_ atmosphere with 90% humidity. The cultures were pulsed with [^3^H] thymidine (0.5 *μ*Ci/well) for the last 18 to 24 h of incubation, cells were harvested, and radioactivity of [^3^H] thymidine incorporation was counted using a scintillation counter (Perkin Elmer Life Science, Boston, MA). [^3^H] Thymidine uptake was expressed as the mean counts per minute (c.p.m.) of sextuplicate wells.

### 2.7. Determination of Cytokine Production

Plasma interleukin IL-6 levels were used as a means of evaluating systemic inflammation and were quantified with commercial ELISA kits (R&D Systems Inc., Minneapolis, MN, USA). Interferon IFN-*γ* and IL-2 levels in supernatants were used in analysis of T helper lymphocyte subclasses Th1 cytokines and IL-10 as a Th2 cytokine. Cytokines were also quantified with commercial ELISA kits (R&D Systems Inc.). Plasma HMGB1 levels were quantified with a commercial ELISA kit (IBL Int. Corp., Toronto, Canada).

### 2.8. Liver Damage Assessment

To assess hepatic function and cellular injury following PF, plasma levels of alanine aminotransferase (ALT) and aspartate aminotransferase (AST) were measured using the Dri-Chem 7000 Chemistry Analyzer (Heska Co., Loveland, CO, USA, slides from FUJIFILM Corp., Japan).

### 2.9. Western Blotting Analysis

Western blot analysis was used to assess plasma HMGB1 level in whole plasma. 0.2 *μ*L plasma in a 20 *μ*L system was separated by SDS-PAGE and transferred onto a nitrocellulose membrane. Lipopolysaccharides-stimulated macrophages were used as positive controls. The membrane was blocked for 1 h in PBS-Tween (0.1%) with 5% milk, followed by immunostaining with optimized dilutions of a polyclonal rabbit anti-mouse HMGB1 antibody (1 : 1,000) in 1% milk in PBS-Tween overnight at 4°C. The membrane was then incubated in PBS-Tween + 1% milk containing HRP-conjugated goat anti-rabbit IgG (Pierce) for 1 h at room temperature. Chemiluminescent signal was developed using SuperSignal West Pico reagents (Pierce) and imaged on X-ray film. Analysis of area values complementary to western blot results was performed using ImageJ software (US National Institutes of Health, Bethesda, MA, USA) [24, 25].

### 2.10. Statistical Analysis

The data are expressed as mean ± standard error of the mean. Comparisons between groups were performed using one-way analysis of variances (ANOVA) and a post hoc Tukey test, and differences were considered significant at *P* < 0.05. The individual studies described in the results section are representative of at least three independent studies.

## 3. Results

### 3.1. Peripheral Tissue Trauma Elicits an Early Inflammatory Response and a Late Attenuated T-Cell Response

To examine the changes of immunoinflammatory response across time after acute peripheral tissue trauma, we examined circulating cytokine mediators, T-cell proliferation, and* in vitro* Th1/Th2 cytokines production at time intervals of 1, 6, 24, 48, and 72 h after PF. Anaesthetized mice that received no experimental manipulation were used as uninjured controls. PF is a model of peripheral tissue trauma, which incorporates all the elements of this injury type including the soft tissue and fracture components [22, 23]. We had previously identified the early inflammation in PF mice, which showed a similar reproducible response to that found with the bilateral femur fracture model [26, 27]. Here we further found that the PF-induced early inflammatory response, which was assessed using systemic IL-6 levels (Supplementary Figure  1A available online at ), was upregulated early with its peak at 1 h and recovered to normal levels by 24 h after trauma. As expected, hepatic injury, assessed by circulating AST and ALT levels, was elevated by 6 h and recovered to normal levels by 48 h (Supplementary Figures  1B and  1C).

The time course of splenocyte proliferation in response to stimulation with con A in cells isolated from PF mice is shown in Supplementary Figure  2A. Splenocyte proliferation was depressed by 48 h after injury and recovered to normal levels by 72 h, when compared with responses of cells from uninjured controls. Next we assessed the Th1/Th2 cytokines released by the splenocytes from PF mice at 48 h after trauma. The* in vitro* release of cytokines by T-lymphocytes is shown in Supplementary Figure  2B. The production of Th1 (IFN-*γ* and IL-2) cytokines by splenocytes was significantly lower in PF mice than in controls, while the production of Th2 (IL-10) cytokines was significantly higher in PF mice. The T-cell proliferative responses and Th1/2 shift induced by anti-CD3 were similar to those induced by con A in these groups (data not shown). Thus, these results suggest that peripheral tissue trauma elicits an early inflammatory response and a late attenuated T-lymphocyte response.

### 3.2. Peripheral Tissue Trauma Elicits Mobilization and Accumulation of CD11b^+^Gr-1^+^ MDSCs in the Spleen

**Figure 1:**
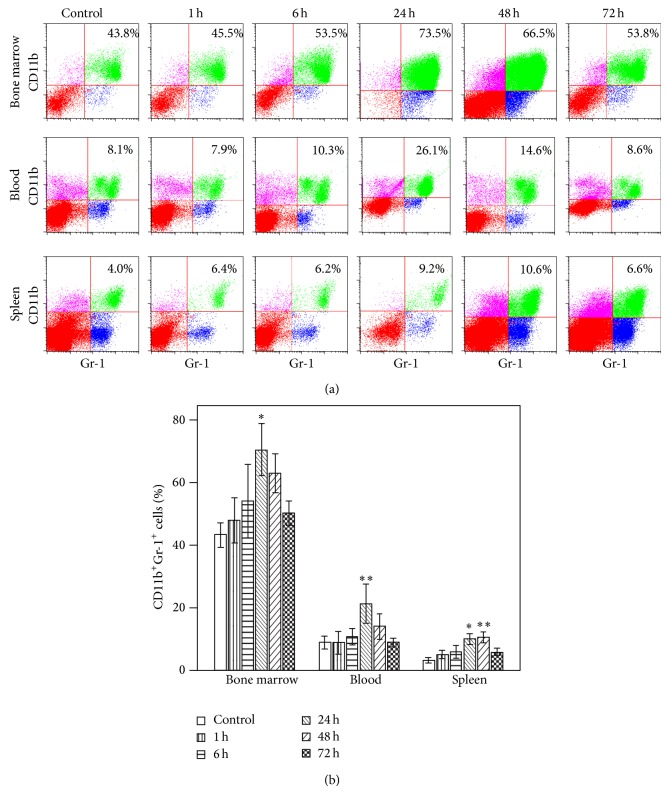
Time course of the changes in accumulation of CD11b^+^Gr-1^+^ myeloid-derived suppressor cells (MDSCs) after pseudofracture (PF) in mice. (a) Representative flow cytometric analysis and (b) graphic analysis of the percentages of CD11b^+^Gr-1^+^ cells in bone marrow cells, blood leukocytes, and splenocytes in mice at time points after injury. ^*^ *P* < 0.05; ^**^ *P* < 0.01 versus control time point. Data represent means ± SEM; *n* = 3 mice per time point.

Recently, the accumulation of MDSCs in the spleen has been reported to play a key role in the immunosuppression after physical injury [7]. Therefore, we determined whether peripheral tissue trauma has any effect on the expansion of CD11b^+^Gr-1^+^ MDSCs in bone marrow, blood, or spleen across 3 days after injury. As can be seen in Figure 1, PF significantly increased the percentages of MDSCs in all 3 lymphocyte compartments. The increased percentage of CD11b^+^Gr-1^+^ MDSC in bone marrow (73% versus 43%), blood (26% versus 8%), and spleen (9% versus 4%) is significant by 24 h after injury. The greatest increases were measured at 48 h with a 3-fold increase seen in the spleen. All the changes in MDSC percentages in different lymphocyte compartments returned to baseline by 72 h, the last time point assessed.

### 3.3. Role of HMGB1 in Peripheral Tissue Trauma-Immune Changes

**Figure 2:**
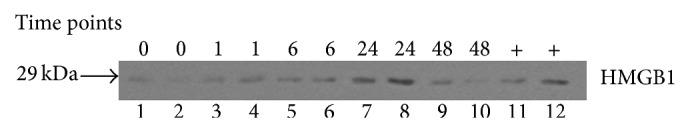
Time course of plasma HMGB1 level after pseudofracture (PF). Western blot of HMGB1 in plasma at time points after PF. LPS-stimulated macrophages were used as positive controls (lanes 11-12). Control mouse was used as time 0. *n* = 3 mice per time point.

Circulating HMGB1 levels were estimated using western blot analysis and further quantified by HMGB1 ELISA. The time course study showed that plasma HMGB1 level increased at 1 h, peaked at 24 h, and remained partially elevated at 48 h after injury (Figure 2, Supplementary Table  1, Supplementary Table  2). This finding is consistent with others that showed elevated circulating HMGB1 levels after blunt trauma [28, 29].

It was reasoned that this HMGB1 elevation might interfere with the immune function after injury. Using a neutralizing anti-HMGB1 monoclonal antibody, 2G7, we next examined the role of elevated HMGB1 on the deleterious effects of trauma on immune system.

**Figure 3:**
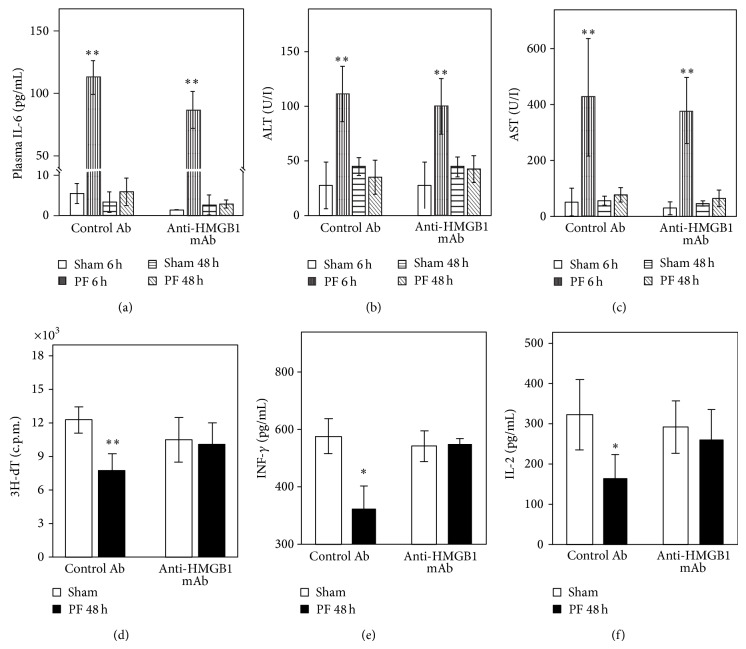
Anti-HMGB1 neutralizing mAb ameliorates attenuated T-cell responses at late time points, but not the early inflammatory responses or hepatic injury after pseudofracture (PF). Plasma IL-6 (a), ALT (b), and AST (c) at 6 h and 48 h after PF in control and HMGB1 Ab-treated mice. Splenocytes were isolated for T-cell proliferation in response to con A (d) and Th1/2 cytokines production ((e), (f)) at 48 h after PF. ^*^ *P* < 0.05; ^**^ *P* < 0.01 versus corresponding uninjured control group. Data represent means ± SEM; *n* = 6–8 mice per group.

The increased plasma IL-6 levels at 6 h, which return to baseline by 48 h after PF, were seen in both the control mAb- and the anti-HMGB1 mAb-injected mice (Figures 3(a)–3(c)). However, the attenuated T-cell proliferation and Th1 cytokine responses observed in injured mice that received control mAb remained intact in anti-HMGB1 mAb-injected mice (Figures 3(d)–3(f)). Thus the impaired T-cell function appears to be associated with the elevated HMGB1 after peripheral tissue trauma.

**Figure 4:**
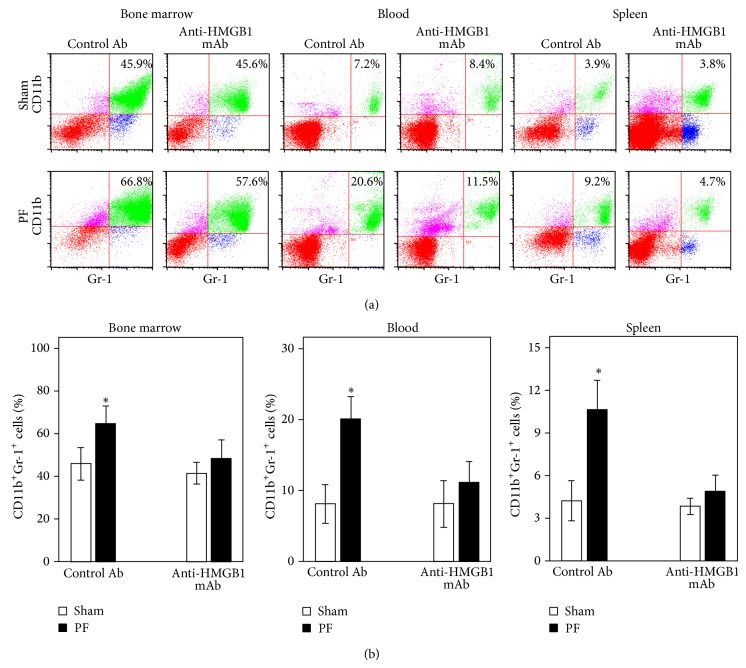
Anti-HMGB1 neutralizing mAb attenuates the expansion of CD11b^+^Gr-1^+^ MDSCs at 48 h after pseudofracture (PF). (a) Representative flow cytometric analysis and (b) graphic analysis of CD11b^+^Gr-1^+^ cell frequencies in bone marrow, blood, and spleen in control and anti-HMGB1 Ab-treated mice at 48 h after PF. ^*^ *P* < 0.05 versus corresponding uninjured control mouse group. Data represent means ± SEM; *n* = 6–8 mice per time point.

Since MDSC can be dissimilarly affected in different lymphocyte compartments, we next examined these possible differences in anti-HMGB1 neutralizing antibody injected mice. Figures 4(a) and 4(b) show the expansion of CD11b^+^Gr-1^+^ MDSCs in bone marrow, as well as their mobilization in blood and accumulation in spleen by 48 h after PF in the control mAb-injected mice. These CD11b^+^Gr-1^+^ MDSC responses to trauma were blocked in the anti-HMGB1 mAb-injected mice. Collectively, our finding that anti-HMGB1 antibody ameliorates the impaired T-cell response and the expansion of MDSC in spleen strongly suggests that trauma-induced immunosuppression requires HMGB1.

## 4. Discussion

Functional alterations have been a consistent immunological finding after severe trauma in patients and in animal models [30–32]. Postinjury aberrations in T-cell function are most consistently found in Th1 cytokine production [31–33]. In addition, the accumulation and induction of MDSCs in the spleen have been suggested recently as a contributor to the immunosuppression that occurs after injury [7, 8]. The main finding of this study is that an HMGB1 neutralizing antibody ameliorates the attenuated T-cell response and increases in CD11b^+^Gr-1^+^ myeloid cells in the spleen two days after peripheral tissue trauma in mice.

In this study, a PF model was used to examine trauma-induced immune imbalance over time. As a model of peripheral tissue trauma, PF incorporates the soft tissue and fracture components of this injury type [22, 23]. We have previously shown that this model induces an early systemic inflammatory response of a similar magnitude to that seen with bilateral femur fracture in mice [22, 23, 26, 27]. However, unlike the bilateral femur fracture model, the PF model does not involve breaking the native bones. Thus, the mice can be recovered, allowing for late posttraumatic survival studies. Here, we show that PF results in a depression in immune functions such as cell proliferation and cytokine production in splenocytes, similar to that seen in other trauma models [34, 35]. Therefore, we conclude that this PF model is useful for assessing the immune changes following peripheral tissue trauma.

This study and our previous findings [23] show a significant decrease in the mitogen-induced proliferation in mice spleen at 48 hours after PF compared with uninjured controls. In addition, we also see the expected suppression of Th1 cytokine production and increases in representative Th2 cytokines at 48 h released by activated splenocytes isolated from injured animals, consistent with previous reports [31, 33]. However, this is in contrast to the findings of Chaudry et al. [36] who showed splenic and peritoneal macrophages, as well as T-cell functions depressed in male animals early, within 30 min after trauma-hemorrhage. This discrepancy may be due to a difference in the severity of the trauma models; the work by Chaudry et al. focused more on a model of systemic ischemia and reperfusion, whereas our model focuses on peripheral tissue injury.

Myeloid-derived suppressor cells have gained significant attention in recent years for their role in acute inflammatory processes such as those that occur during sepsis and trauma [9, 17, 37]. We therefore asked whether peripheral tissue trauma was associated with expansion of CD11b^+^Gr-1^+^ cells in the bone marrow, as well as their mobilization to the circulating blood and accumulation in the spleen at various time points across 3 days after injury. We found a significant increase in numbers of CD11b^+^Gr-1^+^ MDSCs in bone marrow, in blood, and in spleen at 24 h after trauma, while by 48 h only the increase in spleen remained. This study identifies a significant accumulation of cells with MDSC markers (CD11b^+^Gr-1^+^ myeloid cells) early after trauma, yet this study does not directly show their immunosuppressive capacity. However, the findings are in agreement with a previous study [7], where trauma-induced CD11b^+^Gr-1^+^ MDSCs, which show functional immunosuppressive capacity, also exhibit significant early accumulation, importantly highlighting the timeline differences from other disease models. For example, sepsis expands this population in days while tumor implantation does so in weeks [9, 10].

Accumulating evidence has shown that MDSCs regulate immune responses in infections, acute and chronic inflammation, trauma, and surgical sepsis, although initial observations and most of the current information regarding the role of MDSCs have come from studies about cancer [37]. In many mouse tumor models, as many as 20–40% of nucleated splenocytes are represented by CD11b^+^Gr-1^+^ MDSCs (in contrast to the 3–5% seen in normal mice in the present study). It has been demonstrated that the expansion of MDSCs is influenced by several factors that are produced mainly by tumor cells or by activated T-cells. However, in contrast to the prolonged expansion shown in cancer and chronic infection [9, 17], the expansion of MDSCs in the setting of acute trauma is transient. This transient CD11b^+^Gr-1^+^ myeloid population may possibly mediate the suppressive functions that are characteristic of MDSCs. However, because the acute conditions are short-lived, the suppressive functions of this transient population may have a minimal impact on the overall immune response. Therefore, these cells may function as important “gatekeepers” that prevent pathologic immune-mediated damage. Indeed, CD11b^+^Gr-1^+^ MDSCs have been demonstrated to infiltrate the spleen and suppress T-cell function and increase susceptibility to* Listeria monocytogenes* infection in a trauma model [7, 38]. Furthermore, some evidence suggests that MDSCs can induce expansion of regulatory T-cells, another suppressive regulator of the immune system [39]. Interestingly, Li et al. [16] have shown that an MDSC expansion after surgical trauma in the setting of cancer promoted peritoneal metastasis. Collectively, it is reasoned that this transient expansion of MDSCs is involved in the immune imbalance to trauma.

HMGB1 levels are known to be elevated early following trauma in humans [28, 29] and remain elevated. Prompted by the pattern of elevated HMGB1, we hypothesized that treatment with a neutralizing anti-HMGB1 monoclonal antibody would impact the immunosuppression at late posttraumatic term. When measurements were made at 48 h after injury, treatment with two doses of s.c. anti-HMGB1 antibody provided significant protection against the development of trauma-induced immunosuppression including the depressed T-cell response and accumulation of MDSCs in spleen. The precise mechanisms for protective effects of anti-HMGB1 mAb on trauma-induced immune dysfunction in the spleen remain to be established. Excessive production of early proinflammatory mediators could cause and enhance subsequent immunosuppression. Here we showed that early inflammation manifested by circulatory IL-6 levels was unresolved while late immune suppression was ameliorated in the HMGB1 mAb treated mice after trauma. In the context of the present study, it is worth pointing out that the mechanistic relationship between the early hyperinflammatory and the posttraumatic immunosuppression is not known. These two components comprise the current paradigm of the host response to injury, which has been widely accepted over the last 2 decades. This paradigm is directly based on the clinical manifestations of trauma and is supported by an immense body of literature, which has catalogued the complex response to injury that disrupts the immune system homeostasis [40–44]. Current changes to this paradigm suggest an altered timeline, due to recent findings by Xiao et al. [45] and others [46] which have reported that hyperinflammatory and hypoinflammatory responses were concurrent following blunt trauma in humans. However a definitive understanding of the mechanistic correlation of these two components remains to be determined. Our previous work [47] has highlighted the distinct hyperinflammatory and immunosuppressive responses to trauma; we identified that even though Toll-like receptor (TLR) 4 contributed to both responses, TLR9 contributed only to immunosuppressed T-lymphocyte responses after peripheral tissue injury. It is of note that HMGB1 is one of several known ligands for both of these receptors. Therein, these findings suggest that hyperinflammatory response and immunosuppression are not always mechanistically linked and also indicate that HMGB1 may use some alternate mechanisms following tissue trauma alone that lead to the immunosuppression. HMGB1 is also known to mediate different functions dependent on the redox status of the protein and its binding targets [48]. Whether the antibody used in our studies selectively blocks HMGB1 function is not known.

Several inflammatory mediators, such as IL-1 [17], IL-6 [18], TNF-*α* [19], IFN-*γ* [19], and proinflammatory S100 proteins [49], have been demonstrated to be able to drive the expansion of MDSC [20, 21]. Additionally, there is abundant evidence to support a molecular chaperon property of HMGB1 through enhancement of presentation of bound mediators to their cellular receptors. This notion is supported by many studies, showing that HMGB1 binds IL-1, IL-6, TNF-*α*, or IFN-*γ* for an enhanced cytokine-induced effect [50–56]. Thus, HMGB1 may drive the expansion of CD11b^+^Gr-1^+^ MDSCs through binding with these mediators in a lymphoid compartment after trauma. Indeed, this deduction is supported by our results showing that treatment with anti-HMGB1 antibody ameliorated the expansion of CD11b^+^Gr-1^+^ MDSCs and the accumulation in spleen, in the late posttraumatic term. It is possible, of course, that HMGB1 is not directly involved in the expansion of MDSCs, but rather responsible for the release of mediators, which in turn drive the expansion following trauma. Although the molecular mechanisms responsible for HMGB1 in the expansion of CD11b^+^Gr-1^+^ MDSCs and in the CD11b^+^Gr-1^+^ MDSCs-mediation of T-cell suppression remain to be determined, our data suggest that expansion of CD11b^+^Gr-1^+^ myeloid cells in mice after peripheral tissue injury requires HMGB1.

Although this study demonstrates new insights regarding the role of tissue-derived HMGB1 in the response of T-cells and the expansion of CD11b^+^Gr-1^+^ MDSCs to trauma, it has limitations. For example, we may not be measuring the whole of the immune compartment through our current methodology. For example, T-cell numbers were not measured in the thymus or lymph nodes. Ezernitchi et al. [57] have reported that T-cell dysfunction is induced in the spleen, peripheral blood, and bone marrow, but not in lymph nodes, and correlates with elevated levels of MDSC.

In summary, we showed for the first time that treatment with a neutralizing anti-HMGB1 monoclonal antibody can ameliorate attenuated T-cell response and accumulation of CD11b^+^Gr-1^+^ MDSC in spleen in a clinically relevant model of peripheral tissue trauma. It is likely that expansion of MDSCs involves elevated HMGB1 levels and contributes to the immunosuppression after trauma. Our findings suggest that anti-HMGB1 antibodies strategies warrant further evaluation as a therapeutic to reduce infection and MODS after trauma.

## Supplementary Material

Supplementary Figure 1:
*Peripheral Tissue Trauma Elicits an Early Inflammatory Response*. To examine the changes of immunoinflammatory response across time after acute peripheral tissue trauma, we examined circulating cytokine mediators at time intervals of 1, 6, 24, 48, and 72h after PF. Anaesthetized mice that received no experimental manipulation were used as uninjured controls. We found that the PF-induced early inflammatory response, which was assessed using systemic IL-6 levels was upregulated early with its peak at 1 h and recovered to normal levels by 24 h after trauma (Supplementary Figures 1A). As expected, hepatic injury, assessed by circulating AST and ALT levels, was elevated by 6 h and recovered to normal levels by 48 h (Supplementary Figures 1B and 1C).
Supplementary Figure 2:
*Peripheral Tissue Trauma Elicits a Late Attenuated T-Cell Response*. To examine the changes of immunoinflammatory response across time after acute peripheral tissue trauma, we examined T-cell proliferation, and in vitro Th1/Th2 cytokines production at time intervals of 1, 6, 24, 48, and 72h after PF. The time course of splenocyte proliferation in response to stimulation with con A in cells isolated from PF mice is shown in Supplementary Figure 2A. Splenocyte proliferation was depressed by 48 h after injury and recovered to normal levels by 72 h, when compared with responses of cells from uninjured controls. Next we assessed the Th1/Th2 cytokines released by the splenocytes from PF mice at 48 h after trauma. The in vitro release of cytokines by T-lymphocytes is shown in Supplementary Figure 2B. The production of Th1 (IFN-γ and IL-2) cytokines by splenocytes was significantly lower in PF mice than in controls, while the production of Th2 (IL-10) cytokines was significantly higher in PF mice. The T- cell proliferative responses and Th1/2 shift induced by anti- CD3 were similar to those induced by con A in these groups (data not shown).
Supplementary Table 1:
*Circulating HMGB1 levels after Peripheral Tissue Trauma*. Circulating HMGB1 levels were initially estimated using western blot analysis (Figure 2) and further quantified by HMGB1 ELISA (IBL Int.Corp, Toronto, Canada) in Supplementary Table 1. The time course study showed that plasma HMGB1 level increased at 1 h, peaked at 24 h, and remained partially elevated at 48 h after injury.
Supplementary Table 2:
*Circulating HMGB1 levels after Peripheral Tissue Trauma*. Circulating HMGB1 levels were estimated using western blot analysis (Figure 2) and further quantified through analysis of area values complementary to the western blot (Supplementary Table 2) using ImageJ software (U.S. National Institutes of Health, Bethseda, Maryland, USA). The time course study showed that plasma HMGB1 level increased at 1 h, peaked at 24 h, and remained partially elevated at 48 h after injury.
