# Supplementary material for: Anti-HMGB1 Monoclonal Antibody Ameliorates Immunosuppression after Peripheral Tissue Trauma: Attenuated T-Lymphocyte Response and Increased Splenic CD11b+Gr-1+ Myeloid-Derived Suppressor Cells Require HMGB1
Source: Mediators Inflamm. 2015 Jan 29;2015:458626. doi: 10.1155/2015/458626 (PMC4325468; doi:10.1155/2015/458626)
Supplement: Supplementary file 1 — Supplementary Figure 1: Peripheral Tissue Trauma Elicits an Early Inflammatory Response. To examine the changes of immunoinflammatory response across time after acute peripheral tissue trauma, we examined circulating cytokine mediators at time intervals of 1, 6, 24, 48, and 72h after PF. Anaesthetized mice that received no experimental manipulation were used as uninjured controls. We found that the PF-induced early inflammatory response, which was assessed using systemic IL-6 levels was upregulated early with its peak at 1 h and recovered to normal levels by 24 h after trauma (Supplementary Figures 1A). As expected, hepatic injury, assessed by circulating AST and ALT levels, was elevated by 6 h and recovered to normal levels by 48 h (Supplementary Figures 1B and 1C). Supplementary Figure 2: Peripheral Tissue Trauma Elicits a Late Attenuated T-Cell Response. To examine the changes of immunoinflammatory response across time after acute peripheral tissue trauma, we examined T-cell proliferation, and in vitro Th1/Th2 cytokines production at time intervals of 1, 6, 24, 48, and 72h after PF. The time course of splenocyte proliferation in response to stimulation with con A in cells isolated from PF mice is shown in Supplementary Figure 2A. Splenocyte proliferation was depressed by 48 h after injury and recovered to normal levels by 72 h, when compared with responses of cells from uninjured controls. Next we assessed the Th1/Th2 cytokines released by the splenocytes from PF mice at 48 h after trauma. The in vitro release of cytokines by T-lymphocytes is shown in Supplementary Figure 2B. The production of Th1 (IFN-γ and IL-2) cytokines by splenocytes was significantly lower in PF mice than in controls, while the production of Th2 (IL-10) cytokines was significantly higher in PF mice. The T- cell proliferative responses and Th1/2 shift induced by anti- CD3 were similar to those induced by con A in these groups (data not shown). Supplementary Table 1: Circulating HMGB1 levels [file 458626.f1.pdf]

Supplementary Figure 1

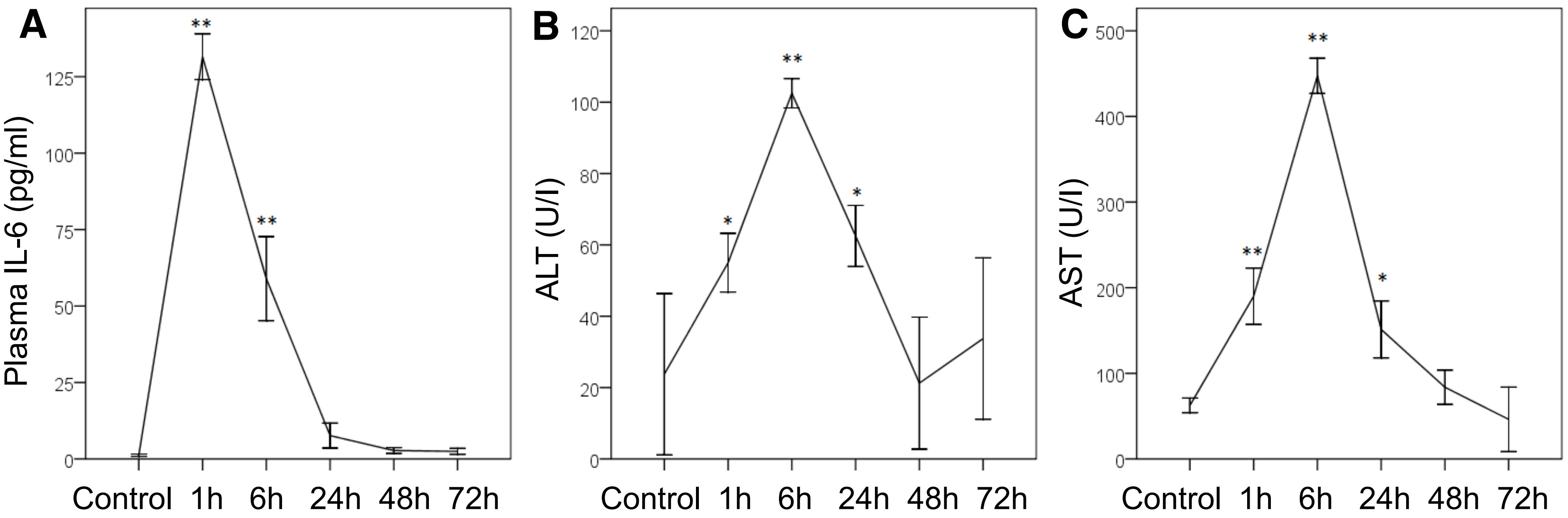

Supplementary Figure 2

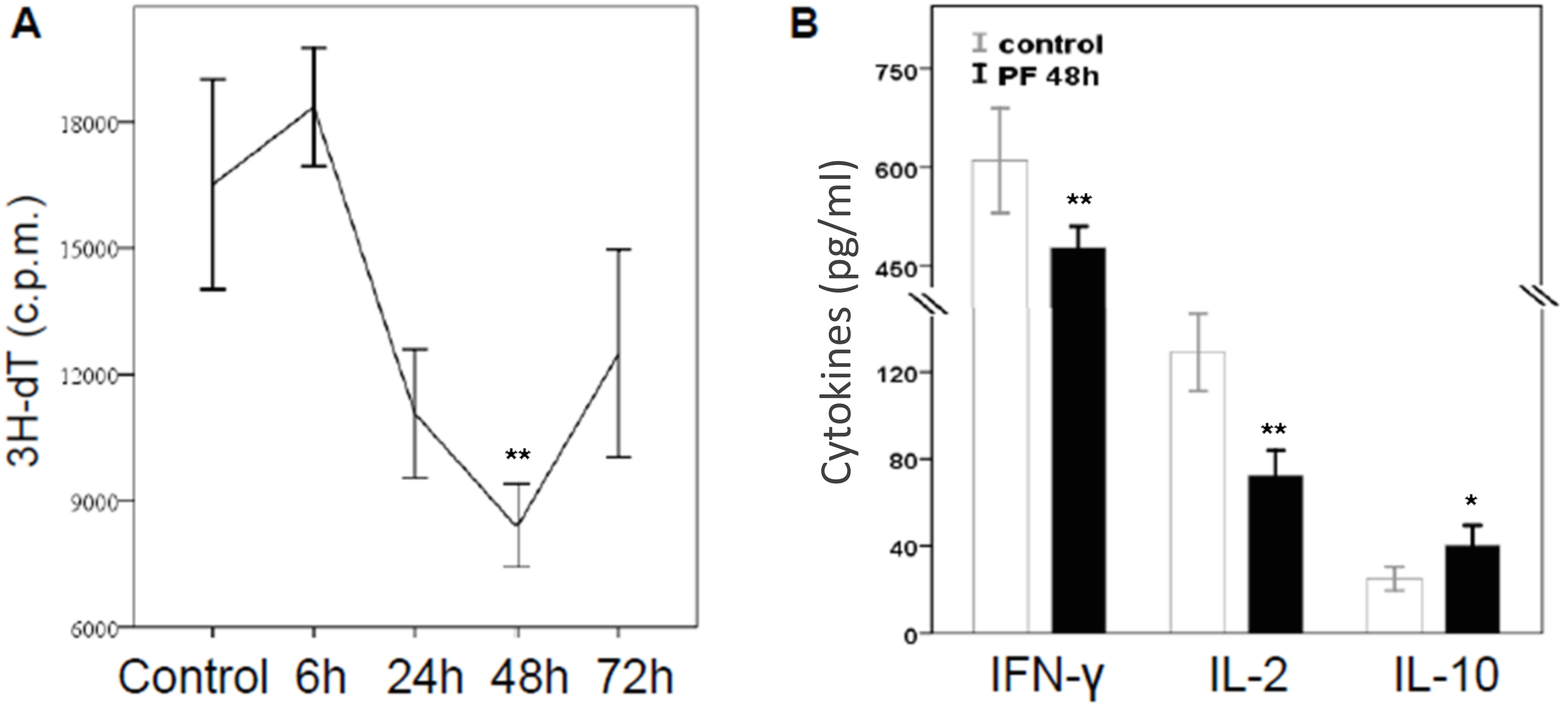

Supplementary Table 1:

Plasma HMGB1 levels peak at 24h after Pseudofracture (PF) injury, measured by HMGB1 ELISA (IBL Int.Corp, Toronto, Canada). Data shown as Mean ± SEM. (n=2 animals/group)

**Group:**	**Control**	**1h PF**	**6h PF**	**24h PF**	**48h PF**
**Plasma HMGB1 (ng/ml)**	15.795 ± 5.34	17.2785 ± 1.58	8.198 ± 2.73	31.9395 ± 18.40	13.7895 ± 1.13

Supplementary Table 2:
Plasma HMGB1 levels peak at 24h after Pseudofracture (PF) injury. Analysis of Area values complementary to Western blot in Figure 2 using ImageJ software (U. S. National Institutes of Health, Bethesda, Maryland, USA). Data = Mean ± SEM. (n=2 Western blot wells/group)

**Group**	**Control = 0h**	**1h PF**	**6h PF**	**24h PF**	**48h PF**	**+ (positive)**
**Western blot wells**	(1, 2)	(3, 4)	(5, 6)	(7, 8)	(9, 10)	(11, 12)
**Area (arbitrary units)**	65 ± 45.9	200 ± 57.9	214 ± 9.8	408.5 ± 27.9	106.5 ± 65.4	290.5 ± 52.7
